# Deep learning-based free-water correction for single-shell diffusion MRI

**DOI:** 10.1016/j.mri.2025.110326

**Published:** 2025-01-17

**Authors:** Tianyuan Yao, Derek B. Archer, Praitayini Kanakaraj, Nancy Newlin, Shunxing Bao, Daniel Moyer, Kurt Schilling, Bennett A. Landman, Yuankai Huo

**Affiliations:** a Department of Computer Science, Vanderbilt University, Nashville, TN, USA; b Department of Neurology, Vanderbilt University Medical Center, Nashville, TN, USA; c Department of Electrical and Computer Engineering, Vanderbilt University, Nashville, TN, USA; d Department of Biomedical Engineering, Vanderbilt University, Nashville, TN, USA

**Keywords:** Diffusion MRI, Free water elimination, Deep learning, Reproducibility

## Abstract

Free-water elimination (FWE) modeling in diffusion magnetic resonance imaging (dMRI) is crucial for accurate estimation of diffusion properties by mitigating the partial volume effects caused by free water, particularly at the interface between white matter and cerebrospinal fluid. The presence of free water partial volume effects leads to biases in estimating diffusion properties. Additionally, the existing mathematical FWE model is a two-compartment model, which can be well posed for multi-shell data. However, single-shell acquisitions are more common in clinical cohorts due to time constraints. To overcome these problems, we proposed a deep-learning framework that focuses on mapping and correcting free-water partial volume contamination in DWI. It utilizes data-driven techniques to infer plausible free-water volumes across different diffusion MRI acquisition schemes, including single-shell acquisitions. In this work, we study the Human Connectome Project Young Adults (HCP-ya), the HCP Aging dataset (HCP-a) as well as Brain Tumor Connectomics Data (BTC). The evaluation demonstrates that it produces more plausible results compared to previous single-shell free water estimation approaches. The proposed method is generalizable through model fine-tuning and b-value re-mapping when dealing with new data. The results have demonstrated improved consistency of properties estimation between scan/rescan data and accuracy in identifying neural pathways, as well as enhanced clarity in the visualization of white matter tracts.

## 1. Introduction

Diffusion magnetic resonance imaging (dMRI) is a noninvasive technique that provides unique in vivo microstructural information, particularly for studying white matter structure and brain connectivity [1–3]. One common application of dMRI is Diffusion Tensor Imaging (DTI), which is clinically utilized for surgical planning [4]. DTI quantifies the three-dimensional movement of water molecules by assuming Gaussian diffusion within a voxel [5]. Key metrics derived from DTI, such as Fractional Anisotropy (FA) and Mean Diffusivity (MD), provide valuable insights into brain microstructure. FA measures the coherence and directionality of white matter tracts, with higher values indicating greater structural integrity and alignment of fibers. In contrast, MD captures the average rate of water diffusion, offering a non-directional measure of tissue density and health. Elevated MD values often reflect pathological changes, such as increased water content from edema or tissue degradation. These metrics enable DTI to support the delineation of neuronal fibers and assessment of brain microstructure, making it a cornerstone in neuroimaging [6].

**Fig. 1.**
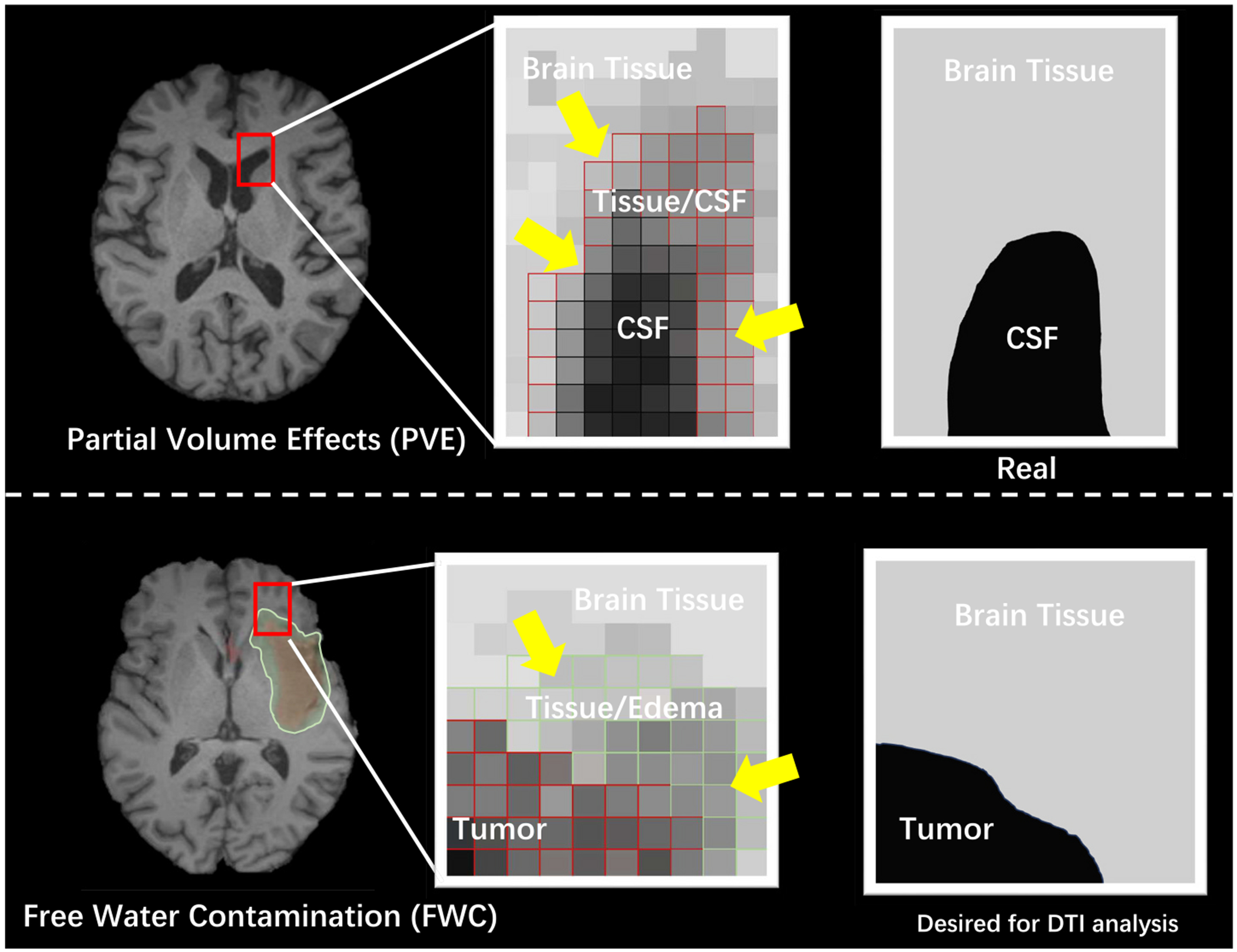
Partial volume effects and free water contamination. In the upper panel, the Free Water partial volume effects (PVE) manifest at the boundary between brain tissue and ventricles filled with cerebrospinal fluid (CSF). The image to the right presents a pristine slice where the distinction between the brain tissue and CSF is sharp and clear. In contrast, the central image reveals partial volume effects in voxels encompassing both CSF and tissue, as indicated by the yellow arrows. In the bottom panel, water molecules diffusing freely (as in edema) tend to reduce the anisotropy measurements because their movement is less restricted and more isotropic compared to water molecules in healthy brain tissue, where diffusion is more anisotropic due to barriers like cell membranes and axonal fibers. The free water contamination can make it challenging to accurately assess white matter integrity and to differentiate pathological changes from changes in water content. The primary objective of the desired algorithm is to extract parameter estimates that are exclusive to the brain tissue within these voxels. (For interpretation of the references to colour in this figure legend, the reader is referred to the web version of this article.)

The single-tensor DTI model assumes a single tissue compartment per voxel, which leads to biased DTI metrics in voxels containing a mixture of white matter and freely diffusing extracellular water molecules [7]. Free water refers to water molecules that are not restricted by cellular barriers and do not experience flow. In the human brain, cerebrospinal fluid (CSF) in the ventricles, around the brain parenchyma, or within lesion edema is considered free water. In the context of dMRI, signals from free water and brain tissue can combine within a voxel due to partial volume effects. When these signals are not explicitly modeled, free water introduces inaccuracies—referred to as “contamination”—that distort the derived diffusion metrics and lead to potential misinterpretations [8]. (Illustrated in Fig. 1.)

This contamination has significant implications for diffusion tractography, where free water can obscure the structural pathways of white matter, and for studying neurodegenerative diseases such as Alzheimer’s disease, Multiple Sclerosis, Parkinsonism, and Schizophrenia. Subtle changes in water diffusion within these conditions often serve as key indicators of disease progression [9]. Accurately addressing these partial volume effects is crucial for reliable interpretation of dMRI data and for isolating tissue-specific diffusion properties.

The Free Water Elimination (FWE) model, as proposed by Pasternak et al. [10], aims to mitigate the adverse impact of CSF partial volume effects on DTI metrics [6]. This model leverages a bi-tensor approach to separate isotropic (free water) and anisotropic (tissue) diffusion components. The initial FWE model requires multiple b-values to distinguish tissue types at the sub-voxel level because water in free and tissue-bound compartments exhibits distinct diffusion behaviors that are more easily disentangled across a range of diffusion weightings [11,12]. Without these multi-shell constraints, the model often becomes ill-posed, making it difficult to estimate free water fractions.

Although mathematical models’ dependence on multi-shell data limits their applicability—since multi-shell acquisitions are not commonly available in clinical practice due to longer scan times [4,12]—in clinical scenarios where single-shell data is more prevalent, direct application of these models is not feasible. To address this limitation, alternative strategies such as spatial regularization methods [9,10] have been proposed to impose smooth constraints and stabilize estimation. Deep learning approaches, like those introduced by Molina-Romero et al. [7], leverage data-driven methods for adapting FWE techniques to single-shell data using multi-layer perceptron. However, traditional deep learning methods [7,37], follow a case-by-case approach and lack generalizability across datasets with diverse acquisition schemes.

In this study, we propose a novel deep learning method [13] for mapping and correcting free-water partial volume contamination in DWI to address these limitations. As compared with the deep learning state-of-the-art (SOTA) method, the proposed deep learning framework can be fed with data simultaneously across a range of acquisition settings (different combinations of b-values within 1000, 2000, 3000 *s/mm*^2^), while targeting the free water fractions generated from Human Connectome Project young adults (HCP-ya) dataset [3,14]. With the combination of dynamic head and spherical convolution, this approach leverages data-driven techniques to reliably infer plausible free-water volumes across different diffusion MRI acquisition schemes, including single-shell acquisitions. During the testing stage, we used only 1000 *s/mm*^2^ shell data (including b0) to evaluate the free water fraction estimation across methods. For extension, we also applied our method to the HCP aging dataset [15] as well as the Brain Tumor Connectomics Data [16,17] (both pre-operative and post-operative). For further evaluation, the test-retest group from HCP-ya is employed for the reproducibility assessment of the proposed free-water elimination method. We also performed tractography and streamline analysis to further demonstrate the plausibility of the proposed method.

## 2. Related work

### 2.1. Free water elimination DTI

Pasternak et al. [10] introduced a bi-tensor model to segregate free water from brain tissue in diffusion MRI. This approach accounts for isotropic (free water) and anisotropic (tissue) components, significantly improving the interpretation of DTI data, particularly in areas with high free water content, such as periventricular zones.

In diffusion MRI, the measured signal is the combined contribution of CSF and tissue (parenchyma) components, where the relative signal contribution of the fast-diffusing component is described by *f*. The free water elimination signal model is expressed as:

(1) $$S=(1-f)\cdot S_{\text{tissue}}+f\cdot S_{CSF}$$

and can be expanded as:

(2) $$S=S_{0}\cdot \left[(1-f)\cdot exp\left(-bg_{i}^{T}D_{\text{tissue}}g_{i}\right)+f\cdot exp\left(-bD_{\text{iso}}\right)\right],$$

where $D_{\text{iso}}$ is the free water diffusivity, $D_{\text{tissue}}$ is the tissue diffusion tensor, and $g_{i}$ represents the diffusion gradient direction. Since $D_{\text{tissue}}<D_{\text{iso}}$, the distinct diffusion characteristics of tissue and free water are better disentangled with multi-shell data acquired at multiple b-values. However, disentangling these volume fractions becomes ill-posed in single-shell diffusion MRI, where diffusion-sensitizing gradients are applied at a single b-value. This limitation renders traditional free water elimination techniques inapplicable without additional constraints [6,12].

### 2.2. Free water elimination for single-shell DWI

In clinical settings, multi-shell datasets are less common due to longer acquisition times, necessitating the development of methods for single-shell data. To address this, Pasternak et al. [10] proposed single-shell strategies, which incorporate spatial constraints and utilize Gradient Descent (GD) to help solve the ill-posed problem. A regularized gradient descent (RGD) method [10,18] has since been implemented as the standard approach for single-shell free water elimination.

While some studies indicate that plausible free water fractions can be obtained using these methods [9], others argue that the resulting maps are unreliable due to the degenerate nature of the model in single-shell acquisitions [6,19,20]. This controversy underscores the challenges of accurately quantifying free water in single-shell data without introducing artifacts or biases that misrepresent tissue properties. Consequently, advanced techniques or complementary strategies are needed to improve the robustness of free water estimation in such scenarios.

### 2.3. Learning-based method

Molina-Romero et al. [7] presented a deep learning framework in their study. This method developed a deep learning methodology for eliminating cerebrospinal fluid (CSF) contamination in diffusion MRI data. This method employs a multi-layer perceptron trained on a synthetic dataset of DWIs to estimate the free water fractions. The network architecture is optimized for processing DWI data, incorporating techniques like dropout and batch normalization to enhance model performance. This approach is particularly significant as it demonstrates the capability of deep learning models to interpret complex medical imaging data, providing a new avenue for more accurate and robust analysis in diffusion MRI, especially in clinical contexts where precise tissue characterization is crucial.

**Fig. 2.**
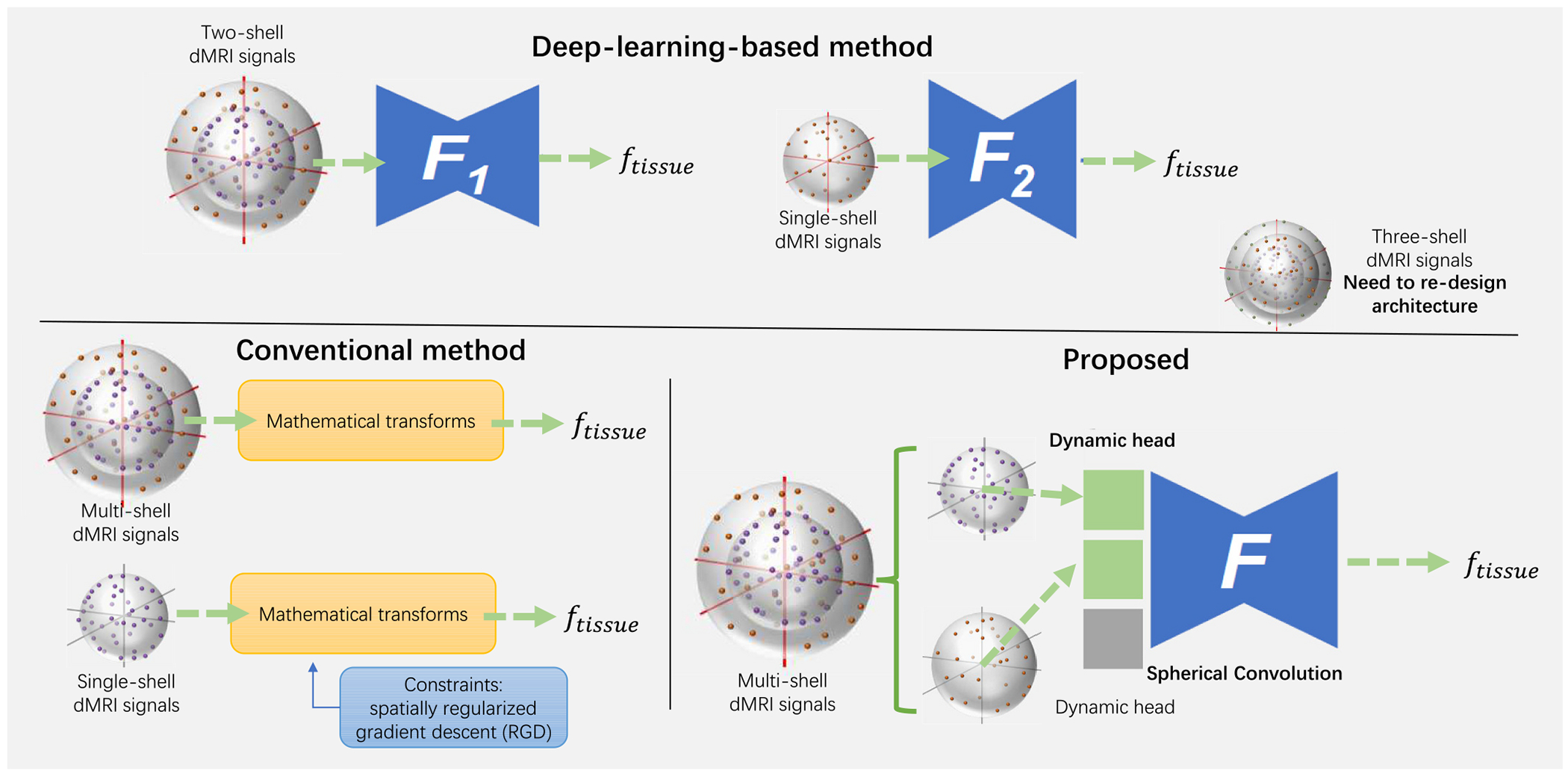
Comparison of methods for free water elimination (FWE) in dMRI. Conventional methods rely on spatially regularized gradient descent (RGD) to impose constraints for solving the FWE problem in single-shell data. However, these methods are often ill-posed for single-shell dMRI and require additional regularization to achieve a stable solution. Previous deep learning-based methods provide accurate model fitting for single-shell and multi-shell data but are typically limited in generalizability due to their case-specific designs (*F*1, and *F*2 represent different network design). These approaches often fail when presented with dMRI data from unseen acquisition schemes. In contrast, the proposed method introduces a unified model (represented as *F*) capable of handling various shell configurations.

However, these deep learning frameworks are not generalizable to new acquisition schemes (as shown in Fig. 2. This complicates applying a deep learning (DL) model to data acquired in different acquisition settings. Our model aims to train a DL framework that can be adapted to an arbitrary number of available dMRI sequences (including multi-shell and single-shell sequences.) By regarding the multi-shell sequences as multi-modality data, a dynamic deep learning framework shall enable input with multiple configurations (single shell, two shells, and three shells, e.g.). During the training stage, the network shall transfer the knowledge between the full shell dMRI with the drop-out self-version, thus enabling the framework to estimate plausible free water fractions for single shell signal input during the inference stage.

## 3. Method

**Fig. 3.**
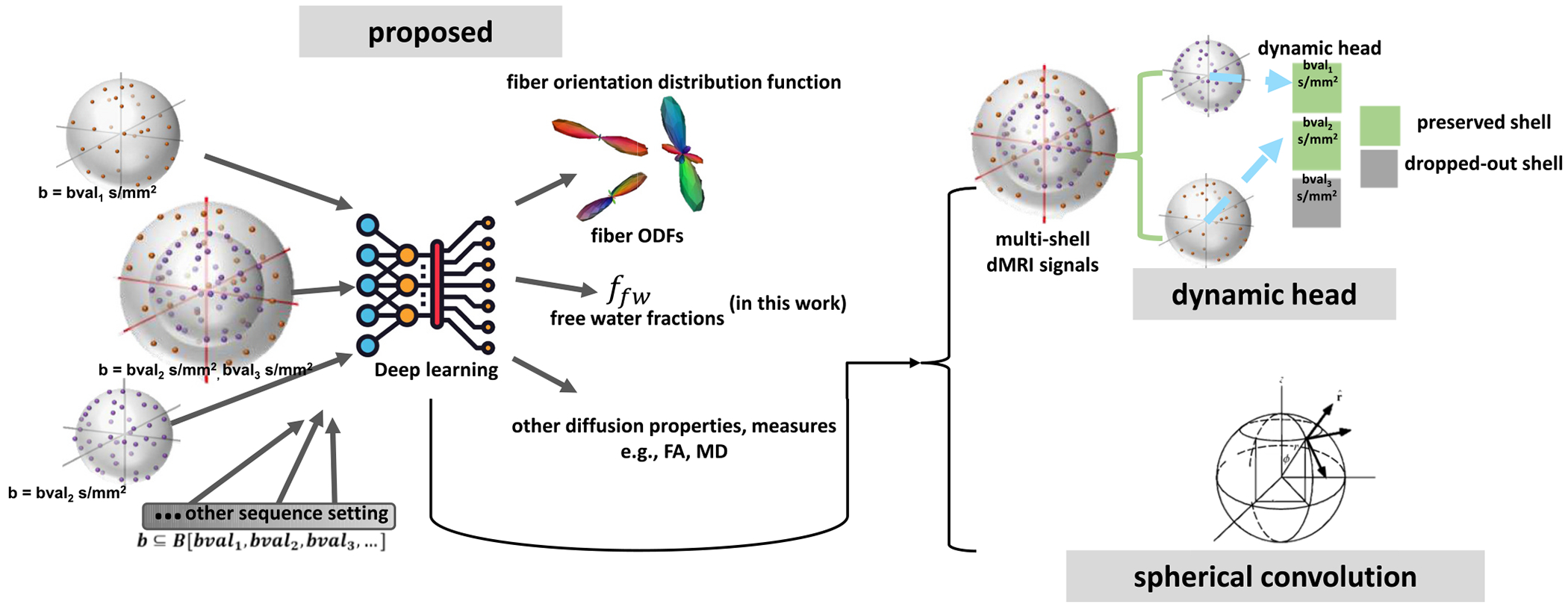
In our study, we aim to design a deep learning framework that integrates signals from both the q-space and radial space as input. This will allow the framework to take in both single-shell and multi-shell diffusion signals (two shells, three shells) that could utilize the powerful data-driven capabilities of deep learning, thus yielding improved accuracy over conventional fitting or modeling of diffusion MRI signals such as free water fractions estimation, ODF estimation and other diffusion measures, like fractional anisotropy, mean diffusivity, etc. we introduce a dynamic head network built on spherical convolutional neural network. The dynamic headings offer a flexible way to handle diverse data types within a single model, adapting its behavior to best suit the input configuration. Using the spherical convolution network serves as a feature extractor to get crucial information from the diffusion MRI signals. Our evaluation of this method focuses on its application to estimate the free water fraction for single-shell DWIs.

To serve the above motivation, we employ a dynamic head (DH) design [13] to handle the multi-shell problem on the three most common b-values: 1000, 2000, and 3000 *s/mm*^2^. Additionally, spherical CNNs were employed to tackle the problem of varying gradient directions on each shell. In this study, we employ free water fraction estimation as our chosen task to perform assessments on both conventional/mathematical models and other deep learning frameworks. The model is shown in Fig. 3.

### 3.1. Dynamic head

**Fig. 4.**
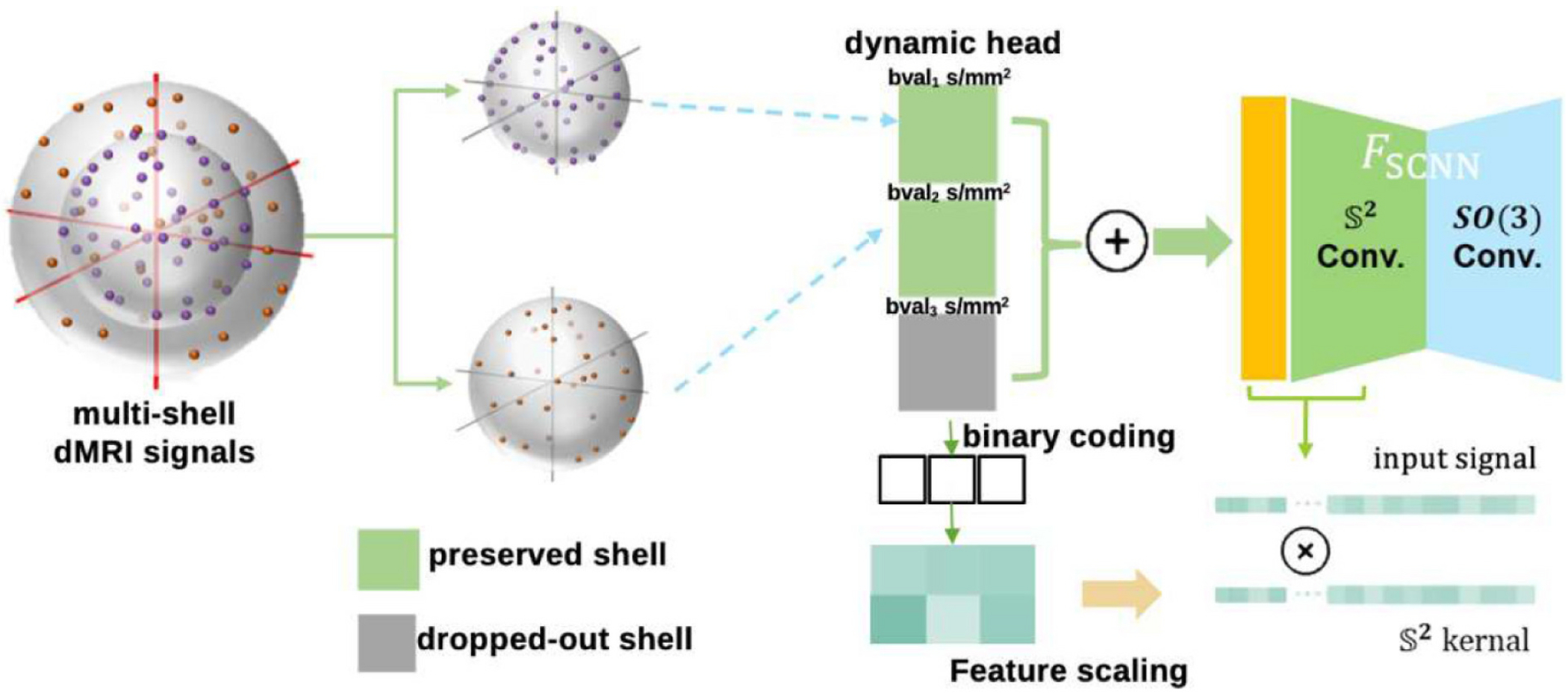
To mitigate the large input variation caused by artificially zero-ed channels, we use the dynamic head to generate the parameters for the first convolutional layer. In our scenario, this feature scaling matrix will adjust the $S^{2}$ convolutional kernel in the spherical convolution part.

A dynamic head design (illustrated in Fig. 4) in multi-modality deep learning [21] offers a flexible way to handle diverse data types within a single model, adapting its behavior to best suit the input modality. In our scenario, we intend to use a dynamic head that allows the neural network to effectively deal with diverse inputs from different shells by adapting its processing mechanism accordingly.

The input is normalized data expanded using spherical harmonics up to degree eight. Note that with *K* shells in our scheme, there are 2*^K^* – 1 configurations. To improve the network expressiveness, we devise a dynamic head to adaptively generate model parameters conditioned on the availability of input shells. We use a binary code $m\in R^{K}$ indicates that *m* is a vector with *K* real-valued entries or components. *K* ∈ [0, 1] that 0/1 represent the absence/presence of each shell. To mitigate the large input variation caused by artificially zero-ed channels, we use the dynamic head to generate the parameters for the first convolutional layer. For a combination of 1000, 2000, and 3000 *s/mm*^2^ in our training configuration, *K* was set to 3 in our study.

### 3.2. Spherical convolution

**Fig. 5.**
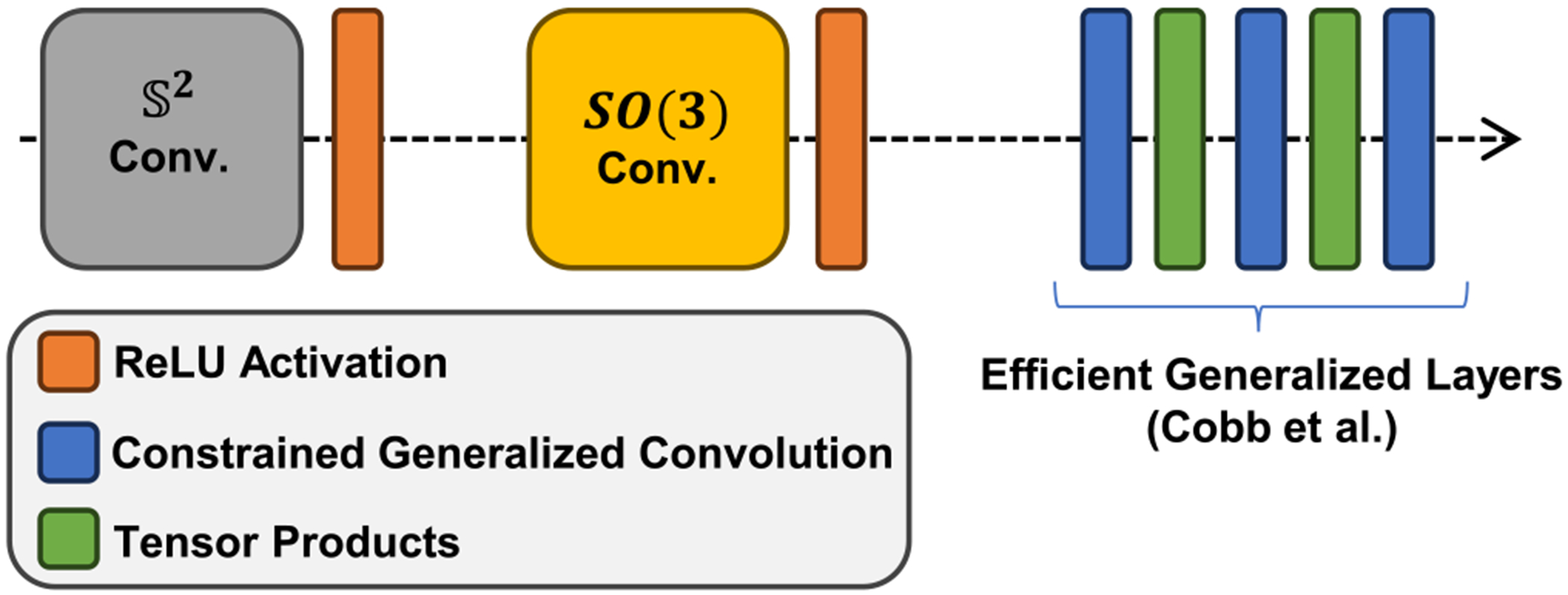
The architecture used for the convolutional base in our spherical-CNN models. The input to the first convolutional layer is a signal on the sphere.

Spherical CNNs are employed to handle variations in gradient schemes and achieve rotational equivariance [22]. After the dynamic filtering, the following architecture (illustrated in Fig. 5) consists of an *S*^2^ convolutional layer and a *SO*(3) convolutional layer. The *S*^2^ layer performs directional convolution on the sphere, lifting the spherical input to the rotation group, while the *SO*(3) layer performs convolution within the rotation group. These layers are followed by three channel-wise activations and two restricted generalized convolutions [22], culminating in a final restricted generalized convolution [22] that maps the features to a rotationally invariant representation. The extracted features are then concatenated and passed through fully connected layers to estimate the free water fraction *f_fw_*.

### 3.3. B-value mapping

Due to differences in the b-values between datasets, we will first match the lower b-values and then proceed to feed into the deep learning framework. Previous work [23] illustrated that diffusion signal has linear decay for values $500s/{mm}^{2}<b_{\text{value}}<1500s/{mm}^{2}$. The equation for the new diffusion signal $S_{b_{\text{new}}}$ is as follows:

(3) $$S_{b_{\text{new}}}=S_{0}*exp\left[\left(b_{\text{new}}/b_{\text{original}}\right)*log\left(S_{b_{\text{original}}}/S_{0}\right)\right]$$

For shells less than or equal to $1500s/{mm}^{2}$, the lowest shell value shall be re-calibrated to $1000s/{mm}^{2}$ to match the training scheme.

### 3.4. DWI estimation from estimated free water fraction

After the *f_fw_* is estimated, the desired FW eliminated signal (interpreted as $\hat{S}$ ) shall be calculated (derived from Eq. (2)) by the following equation:

(4) $$\hat{S}:=S-S_{0}*f_{fw}*exp\left(-bD_{iso}\right)$$

Additional parameter adjustments were applied during the estimation process. A mean diffusivity regularization threshold of 2.7e-3 *mm*^2^. *s*^−1^ was used. This threshold corresponds to the approximate value of free water diffusivity in physiological conditions. If the mean diffusivity exceeds this threshold, the diffusion signal is assumed to originate entirely from free water (i.e., the free water volume fraction is set to 1, and the tissue’s diffusion parameters are set to zero).

## 4. Experiments

### 4.1. Data

We have chosen DW-MRI from the HCP-ya dataset [3,14], which consists of healthy adults aged 22–35 years. A total of 220 subjects were included in this study. The acquisitions had b-values of 1000, 2000, and 3000 *s/mm*^2^ with 90 gradient directions on each shell. The data was preprocessed by the PreQual [26] pipeline with the default settings and Gibbs de-ringing to remove artifacts, including denoising using the Marchenko-Pastur PCA (MP-PCA) technique, intensity normalization, and distortion corrections. Distortion corrections included eddy current[SP]-induced distortion correction, intervolume motion correction, and slice-wise signal dropout imputation. A T1 volume of the same subject was used for brain masks segmented using SLANT [24]. All HCP-ya dMRIs were distortion corrected with top-up and eddy [25]. Of the 220 subjects, 200 were used as training data, 10 as validation, and 10 as testing data. The validation dataset was used during model training to tune hyperparameters and monitor performance, while the testing dataset was kept separate and used only for final performance assessment to ensure unbiased evaluation. To simulate different shell configurations, the data was reorganized to generate two-shell and single-shell datasets (*b*0 shells included). Another cohort from HCP-ya, the test-retest group, includes 10 subjects with scan/rescan DWIs and was employed for test-retest reproducibility assessment. For single-shell free water estimation, only the 1000 *s/mm*^2^ shell from the testing cohort was used.

The diffusion and T1-weighted MRI data of 50 unrelated healthy adults aged 50+ from the Lifespan HCP in Aging (HCP-A) [15] study was used to evaluate the effectiveness of the proposed FWE method on healthy elderly subjects. This dataset spans a wide age range (36–100+years), capturing age-related variability in brain structure and function. Diffusion encoding was acquired with two shells of 1500 and 3000 *s/mm*^2^ (98–99 directions per shell) and 28 b-value = 0 *s/mm*^2^ images interleaved. All datasets were acquired with two phase encoding directions: anterior to posterior (AP) and reversed phase encoding direction (PA). The data was preprocessed by the PreQual [26] pipeline with the default settings and Gibbs de-ringing. The T1 image was registered to the mean b0 image and segmented using SLANT [24]. Of the 50 subjects, 40 were used to fine-tune the model, 5 as validation, and 5 as testing data. The 1500 *s/mm*^2^ shell was processed with a b-value re-mapped to 1000 *s/mm*^2^ before feeding to the deep learning framework.

The third dataset studied is the Brain Tumor Connectomics Data [16,17], which includes both pre-operative and post-operative data. The pre-operative data consists of 11 glioma patients, 14 meningioma patients, and 11 control subjects, while the post-operative data consists of 7 glioma patients, 12 meningioma patients, and 10 control subjects. This dataset provides a clinical perspective, focusing on tumor-related structural changes. The DWI scans were acquired using a multi-shell high-angular resolution protocol at 700, 1200, and 2800 *s/mm*^2^. The data was preprocessed by the PreQual [26] pipeline with the default settings and Gibbs de-ringing. The T1 image was registered to the mean b0 image and segmented using SLANT [24]. Only the 700 *s/mm*^2^ shell was involved in the deep learning experiment and re-mapped to 1000 *s/mm*^2^ before feeding to the deep learning framework. Three DWIs from a subset of unique subjects (patients and controls) were withheld as the testing cohort, ensuring no overlap with subjects whose data was included in the training or fine-tuning datasets. The remaining DWIs from pre-operative and post-operative data were used exclusively for training and fine-tuning.

The target free water fractions are generated using the Free Water Elimination model described in [6,10]. The implementation details of the free water DTI model are provided in [20] and made available through the DIPY library (version 1.70) with its default settings [27]. Since ground truth mappings of free water volume in human data are not available, we use the free water fractions estimated from the full configuration of multi-shell data as the silver standard in this study. This approach assumes that multi-shell HARDI protocols provide a more accurate estimation of free water. Our deep learning-based model is designed to bridge the gap between single-shell data and this silver standard, effectively learning to approximate a more complex method from simpler data inputs.

The target free water fractions are generated using the Free Water Elimination model described in [6,10]. The implementation details of the free water DTI model are provided in [20] and made available through the DIPY library (version 1.70) with its default settings [27]. Since ground truth mappings of free water volume in human data are not available, we use the free water fractions estimated from the full configuration of multi-shell data as the silver standard in this study. This approach assumes that multi-shell HARDI protocols provide a more accurate estimation of free water. While the silver standard is consistent across subjects in terms of processing and assumptions, its accuracy is limited in regions with low signal-to-noise ratio (SNR) or complex microstructural environments where multi-shell models themselves may face challenges. Despite these limitations, the silver standard serves as a practical benchmark for evaluating our model, and its reliability is further supported by the high quality of the datasets and rigorous preprocessing. Our deep learning-based model is designed to bridge the gap between single-shell data and this silver standard, effectively learning to approximate a more complex method from simpler data inputs.

### 4.2. Experimental settings

#### 4.2.1. Deep learning estimation on free water fraction

##### 4.2.1.1. Model-based baseline methods.

The method of Free Water Elimination from Pasternak et al. [10] was implemented as the baseline for the conventional FWE model. When dealing with single shell data, spatially regularized gradient descent (RGD) algorithms [9] applied constraints that are imposed via the time evolution of a gradient flow on a Riemannian manifold [28] to get a unique solution.

##### 4.2.1.2. Deep learning baseline methods.

The ANN proposed by Molina-Romero et al. [7] is performed as a deep learning-based method to extract the free water fraction. We followed their settings by initializing a 4-layer FCN (two hidden layers with *N_b_/*2 and *N_b_/*4 respectively). In our case, *N_b_* is 96. The input size is 96 and one single output unit yields the estimate of free water fractions. We trained the ANN architectures with the training Cohort.

Additionally, the shore basis function [29–31] has been shown to capture the representation of multi-shell dMRI with minimal representation error and ensure the same when modeling single-shell dMRI. Thus, we utilize the shore representation as an additional baseline to assess the representation extracted from the spherical convolution network. We used 6*th* order and regularization constants: 1*e* – 8. The scaling factor is carefully calculated by ζ defined in units of $mm^{-2}$ as $\zeta =1/8\pi^{2}\tau MD$ is calculated based on the mean diffusivity (MD) obtained from the data. We also applied a 4-layer FCN to fit the FW fraction. The input size is 50 (SHORE-based ODF estimates 50 coefficients at 6*th*) and the sizes of the hidden layer are 100, and 50 respectively.

For the implementation of the proposed method, we used a spherical convolution model with the architecture [32]. The first block includes a directional convolution on the sphere that lifts the spherical input onto the rotation group, all parameters for this first convolution block are dynamically generated based on a given prior (dynamic headings). The second block includes a convolution on the rotation group, hence its input and output both live on the rotation group. We then apply a restricted generalized convolution. The same type is used for the following three channel-wise tensor-product activations and two restricted generalized convolutions until the final restricted generalized convolution maps down to a rotationally invariant representation. As is traditional in convolution networks we gradually decrease the resolution, with the spatial spherical harmonic bandlimits/degree [32] (*L*_0_, *L*_1_ , *L*_2_, *L*_3_, *L*_4_,*L*_5_ = (20, 10, 10, 6, 3, 1) and increase the number of channels, with (1, 20, 22, 24, 26, 28). The detail of the efficient generalized layer can be found in [33]. By using *L*_1_ regularization (regularization strength 10^−5^) and applying a restricted batch normalization across fragments (irreducible representations of the rotation group [32,34]), where the fragments are only scaled by their average and not translated (to preserve equivariance).

The RMSE loss was applied as the criterion function during the training process for all deep learning methods, we chose the best-performing models on the validation cohort and compared them against the Pasternak’s with the RGD method on the HCP-ya dataset. After free water elimination, the fractional anisotropy and mean diffusivity are calculated around the whole brain on the test cohort. Histograms of such diffusion properties are generated to assess the effect of regularization on free water estimation across methods.

Additionally, we compared the deep learning models to evaluate the generalizability across different shell configurations. The ANN method for different configurations is trained separately and the input size for the first layer of FCN is adjusted to the corresponding number of gradient directions of input.

#### 4.2.2. Tractography and bundle analysis on free water corrected DWI

For each test subject, we used the MRTrix (version-3.0.3) default probabilistic tracking algorithm of second-order integration over fiber orientation distributions (FODs) for tractography [35]. We generated 10 million streamlines to build each tractogram, seeding, and termination using the five-tissue-type mask. We allowed backtracking. Tracts and binary segmentation masks for each bundle are generated using Tract-Seg [36]. Further specific bundle analyses were performed for different datasets to assess the effectiveness of free water correction of the proposed method.

## 5. Results

### 5.1. Deep learning estimation on single shell DWIs

The RMSE with generated free water fractions and the silver standard is calculated and shown in Table 1. From the table, the deep learning methods outperformed other methods on single-shell data in terms of RMSE for predicting *f_fw_*. Notably, the RMSE of our proposed method is even lower than the scan-rescan variability observed in the high-quality HCP dataset, which demonstrated an RMSE of 0.0354. This highlights the robustness and precision of our model compared to both conventional methods and other deep learning approaches.

### 5.2. Deep learning estimation on heterogeneous configuration DWIs

In Table 2, we evaluated different deep learning pipelines’ generalizability across different shell configurations. To be specific, the ANN method has 7 independent models for different subsets of the 1000, 2000, 3000 *s/mm*^2^ scheme. It is worth noting that the SHORE representation has decent performances in multi-shell configuration with our proposed method which proved to enable multi-shell b-value generalizability of the data-driven deep learning approach. Our proposed method outperforms Deep SHORE in a single-shell scheme proving the dynamic head strategy can produce similar feature representations when the information of multiple shells is absent.

Additional experiments have been conducted to evaluate the proposed framework when applying the model (trained only with HCP-YA data) to the HCP-Aging dataset, as shown in Table 4. During fine-tuning, only the parameters of the fully connected layer are trainable, allowing the model to adapt to new datasets while retaining its core structure. The model was assessed on both single-shell and multi-shell configurations, revealing a significant improvement in performance following fine-tuning. This is evidenced by the reduction in RMSE values across all tested configurations. For example, the RMSE decreased from 3.61 to 2.22 for the 1000 (1500) and 3000 *s/mm*^2^ configuration, highlighting the efficacy of fine-tuning in enhancing model accuracy and generalizability.

Notably, the inclusion of high b-value data (e.g., b3000 *s/mm*^2^) offers a potential advantage, as it significantly attenuates the free water signal, leading to clearer tissue characterization. However, high b-value data is known to suffer from reduced SNR, which can negatively impact model performance in less optimal datasets. While the HCP datasets used in this study provide high-quality data with minimal noise, the generalizability of the proposed method to other datasets with high b-value acquisitions (e.g., b3000 *s/mm*^2^) warrants further exploration.

### 5.3. Diffusion properties estimation after FWE on single shell DWIs

To further estimate the effectiveness of free water elimination to microstructural inspection. A cubic patch, in the central of the volume, which shall include the cerebrospinal fluid confined ventricles (50 × 50 × 50) is selected as the region of interest(*ROI*)to calculate the change of FA in the HCP-ya test cohort as shown in Table 3.

**Fig. 6.**
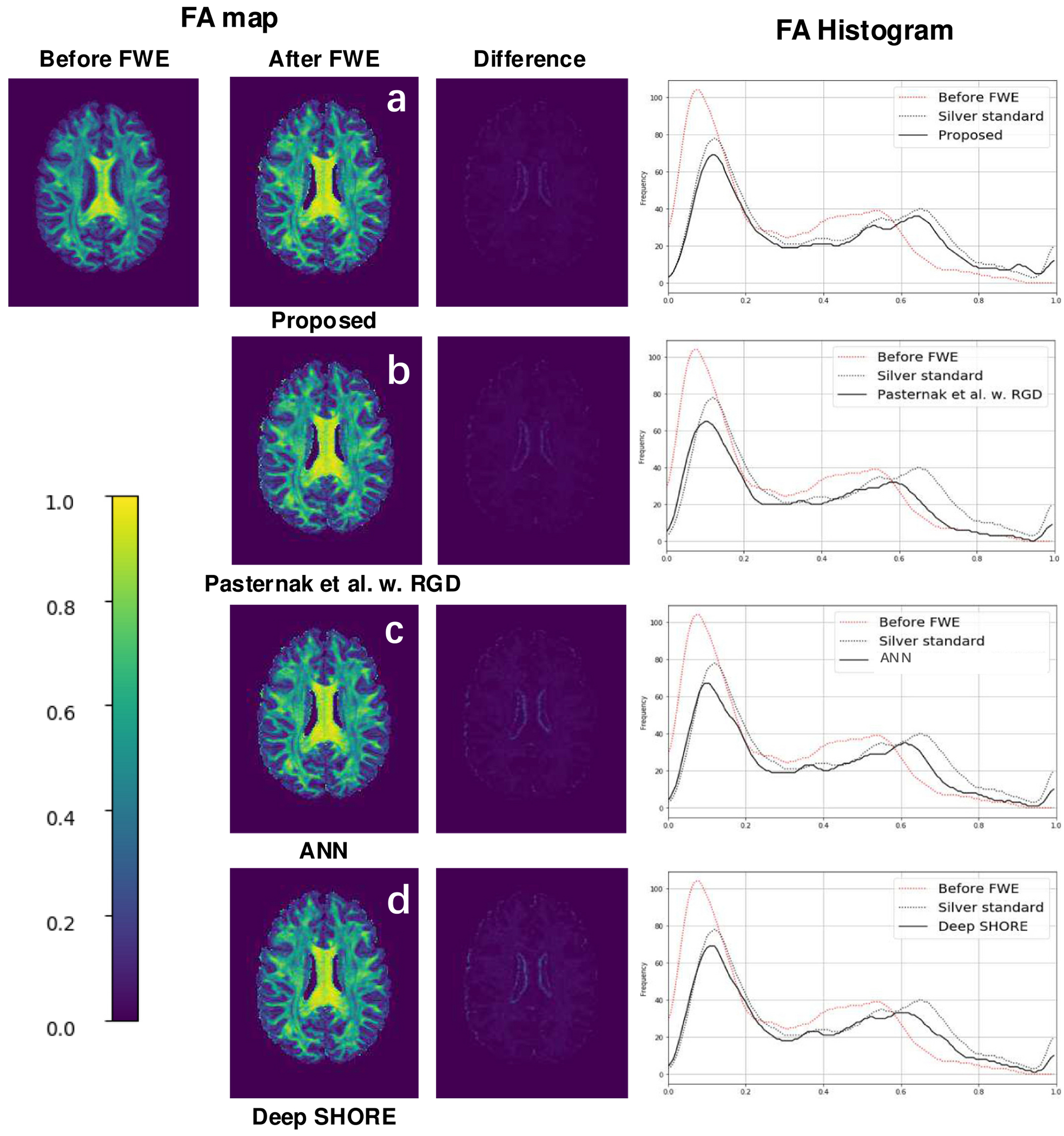
The histogram of the FA is shown before and after FWE from different methods respectively in the top panel. To further assess the transform/prediction, the FA error map with the silver standard is depicted in the bottom panel.

Both our proposed method and the baselines showed similar performance (a boost of FA) as shown in the histogram of Fig. 6. From the table mentioned above, the deep learning methods outperformed other methods on single-shell data on both RMSE of the prediction error of *f_fw_* and the average FA on ROI patches, a visualization of the error with the silver standard is shown in the bottom panel of Fig. 6. Our proposed framework has also reached an average FA of 0.508 on a selected ROI as compared with 0.517 on the silver standard.

**Fig. 7.**
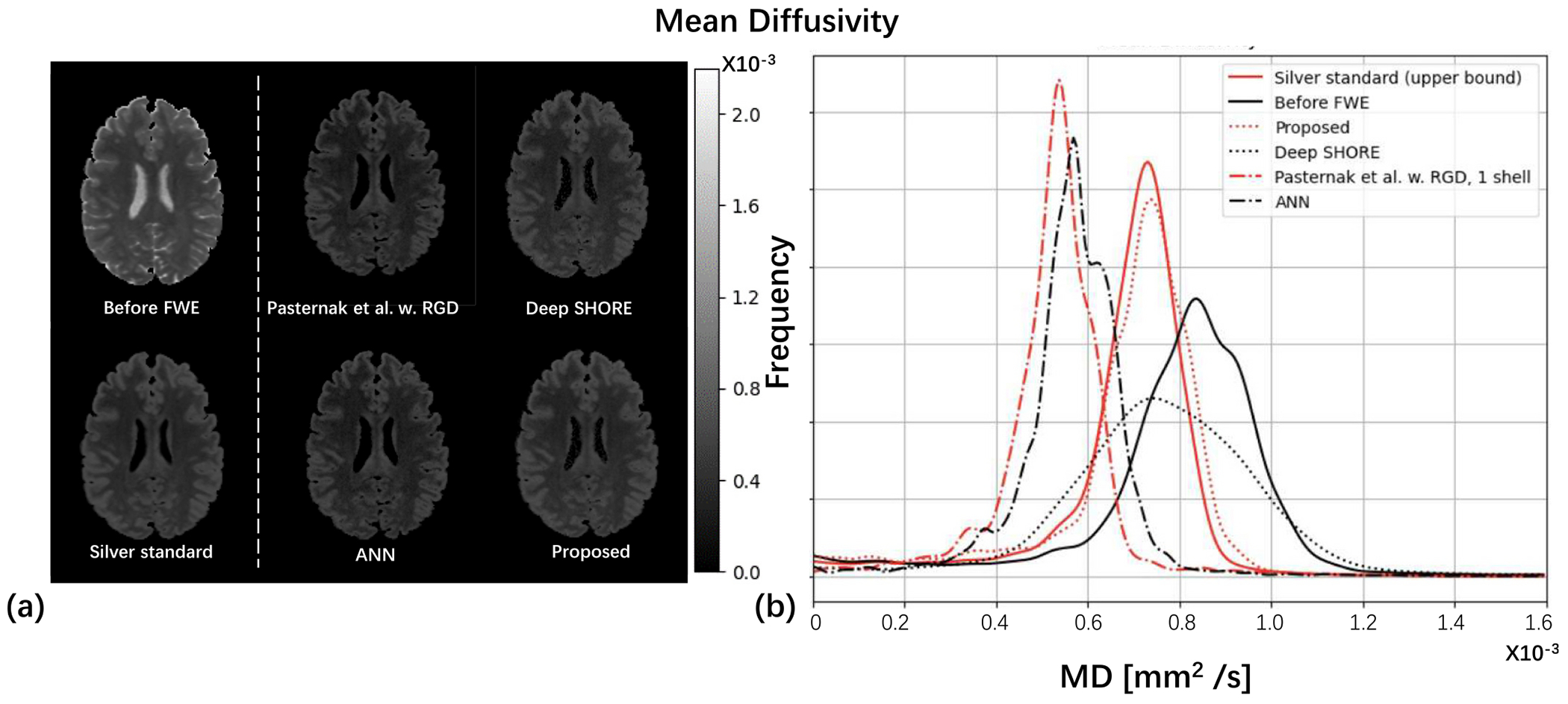
The mean diffusivity of before/after different free water correction methods is qualitatively depicted in (a). To further assess the correction effect, the histogram of MD is shown in (b).

CSF contribution was removed from all the MD maps as shown in the left panel of Fig. 7. However, from the histogram of the right panel of Fig. 7, both ANNs and the Pasternak et al. w. RGD method suffered from over-regularization of MD. Our proposed method has the closest distribution with the MD of the silver standard.

**Fig. 8.**
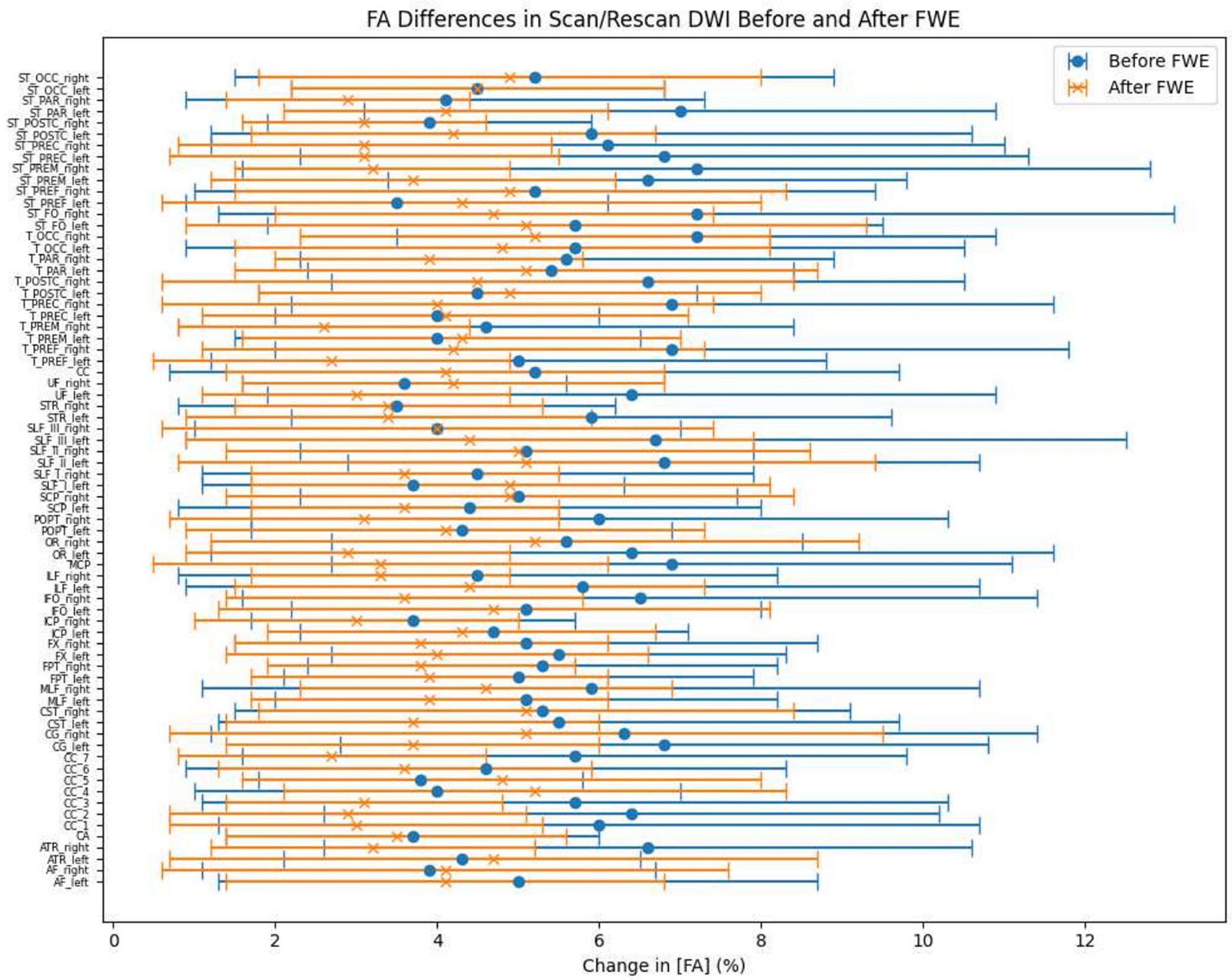
Voxel-wise fractional anisotropy (FA) differences for each of the 72 bundles from the TractSeg bundle map, averaged across regions and HCP-YA test-retest subjects. The FA differences between scan and rescan data were quantified and expressed as percentages. The mean FA differences and standard deviations are presented in a grouped dot plot with error bars. Statistical significance was assessed using a Wilcoxon signed-rank test, yielding a *p*-value of less than 0.05.

**Fig. 9.**
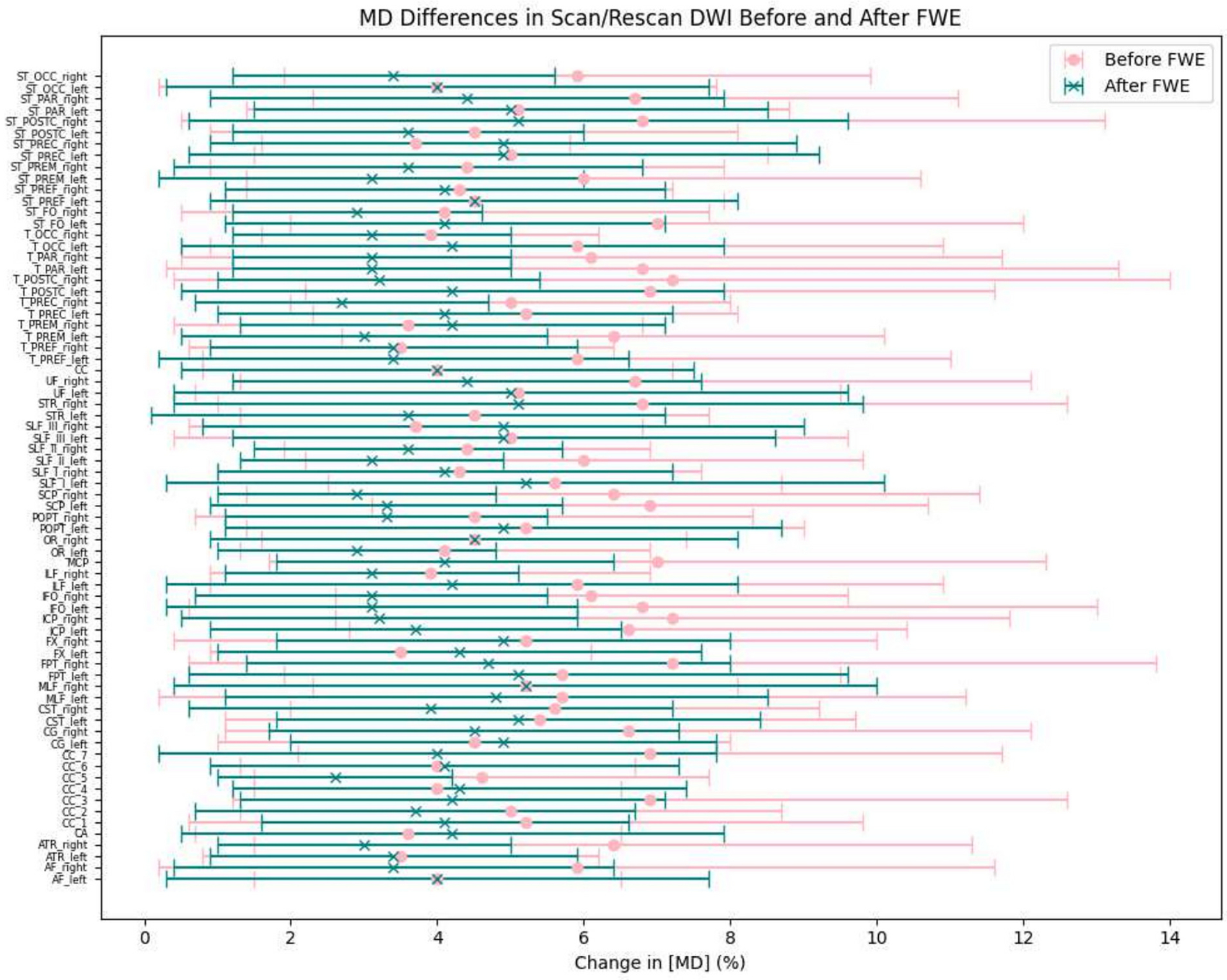
Voxel-wise mean diffusivity (MD) differences for each of the 72 bundles from the TractSeg bundle map, averaged across regions and HCP-YA test-retest subjects. The MD differences between scan and rescan data were quantified and expressed as percentages. The mean MD differences and standard deviations are presented in a grouped dot plot with error bars. Statistical significance was assessed using a Wilcoxon signed-rank test, yielding a p-value of less than 0.05.

Previous work has shown free water elimination improves test–retest reproducibility of diffusion tensor imaging indices in the brain [38], we also assessed the test-retest reproducibility on the HCP-ya test-retest group dataset. Binary segmentation of 72 tracts are generated using TractSeg [36]. The scan/rescan images are registered and the joint voxels for the 72 tracts are selected to calculate the FA, MD voxel-wise and average over regions before and after free water elimination. The differences between the scan and rescan data were quantified and subsequently converted into percentages to facilitate a more nuanced analysis. As shown in Figs. 8 and 9, the grouped dot plot with error bars illustrate that deep learning FWE significantly improves test-retest reproducibility on DTI metrics in brain. The corpus callosum (CC), the corticospinal tract (CST) and the superior longitudinal fascicle (SLF) were selected as ROI and performances metrics on such region has been calculated with an improvement of agreement (reduction of difference) by 4.1 %, 4.3 %, 5.2 % in FA and 6.1 %, 6.3 %, 4.7 % in MD.

### 5.4. Bundle analysis on aging subjects

**Fig. 10.**
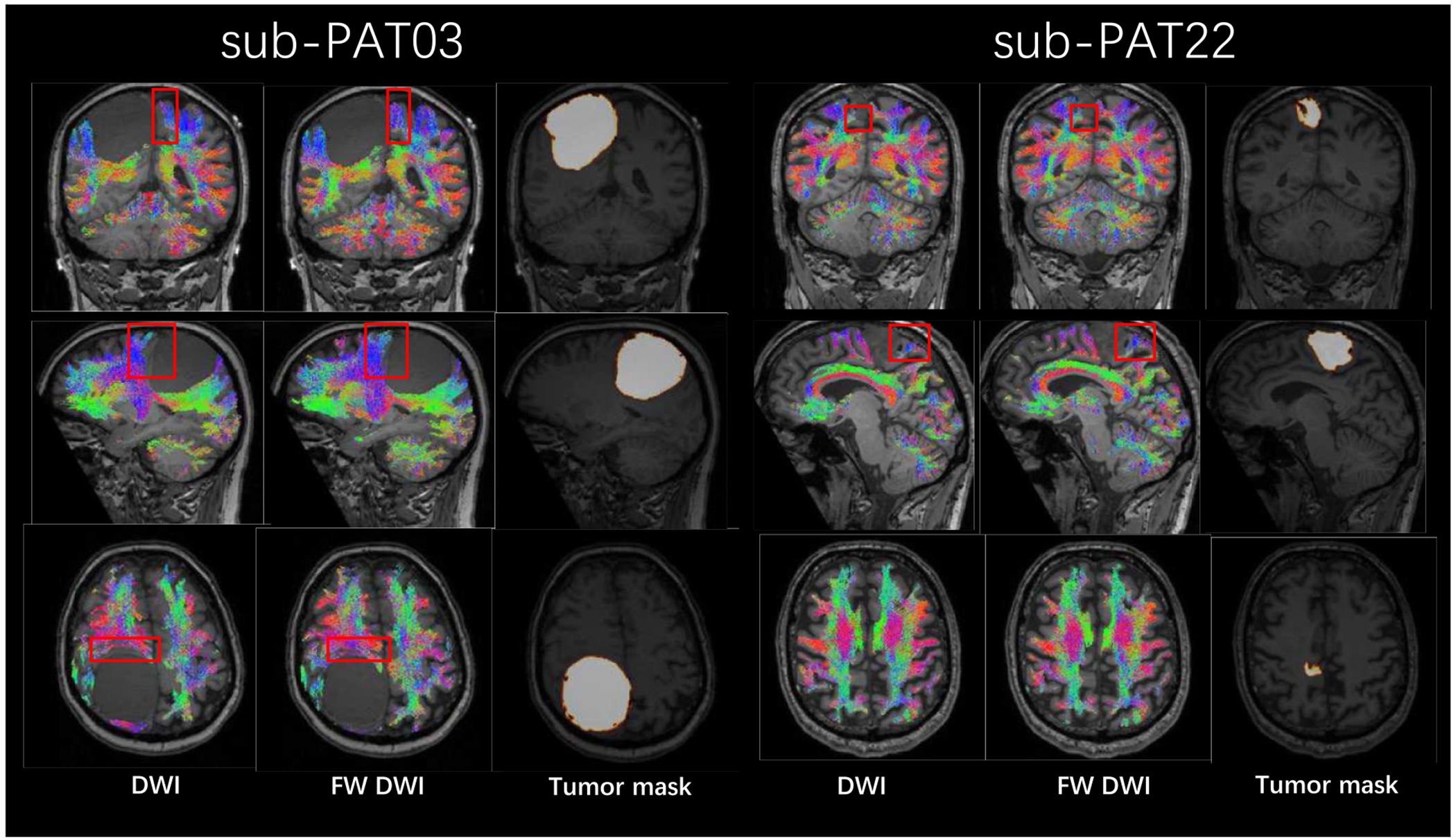
Comparison of tractography results before and after free water elimination for two representative patients: Sub-03, a 60-year-old female with meningioma type I in the parietal brain (tumor size: 78.44 *cm*^3^), and Sub-22, a 53-year-old male with oligodendroglioma type II in the parietal brain (tumor size: 15.16 *cm*^3^). The images display the original DWI (left), FW-corrected DWI (middle), and tumor mask (right). Red boxes highlight regions with notable differences, predominantly in areas affected by tumors, as indicated by the tumor mask. (For interpretation of the references to colour in this figure legend, the reader is referred to the web version of this article.)

**Fig. 11.**
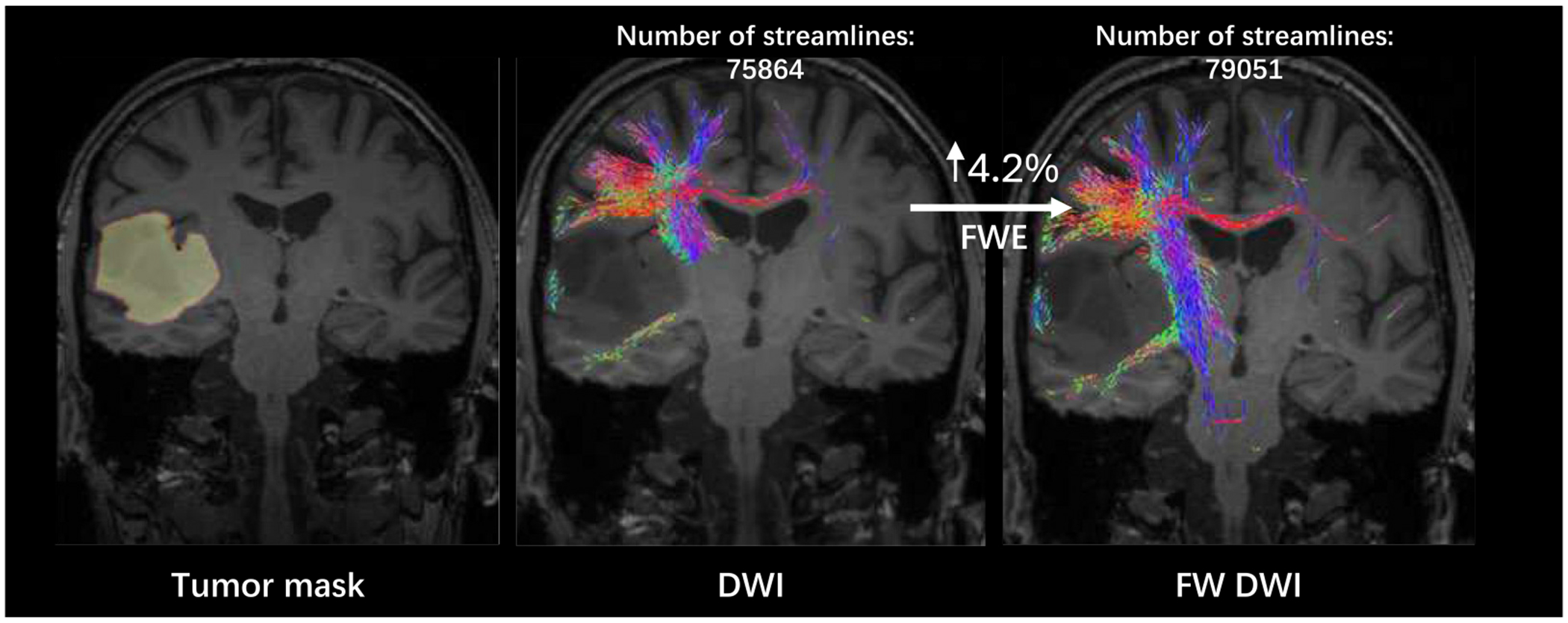
Tractography results for a 61-year-old male patient with anaplastic astrocytoma type III in the temporal brain (tumor size: 59.21 *cm*^3^). The streamlines around the tumor region are displayed, showing a 4.2 % increase in the number of streamlines after free water elimination.

The proposed FWE model is applied to the BTC pre-operative test cohort data after fine tuning with the training cohort. Only the 700 *s/mm*^2^ shell (re-mapping to 1000 *s/mm*^2^) is involved in this section. We performed tractography on both the DWI and FW-corrected DWI, a visualization of the tracts is shown in Figs. 10 and 11 where the bundles around the tumor region are highlighted. The findings demonstrate that most patients exhibit an increase in the extent of the peritumoral region covered by recognizable tracks when free water elimination is employed.

## 6. Discussion

The elimination of free water in Diffusion Weighted Imaging (DWI) analysis is crucial for enhancing the accuracy, specificity, and inter-pretability of imaging results. This process is essential as it allows for a more precise representation of tissue microstructure by mitigating the confounding effects of free water, such as in cerebrospinal fluid or edema, which can otherwise lead to overestimation of tissue diffusion and misinterpretation of pathological states. By employing sophisticated post-processing techniques to separate free water from tissue-bound water signals, free water elimination improves tissue characterization, enhances quantitative analysis, reduces imaging artifacts, and increases the sensitivity of DWI to subtle pathological changes. This refinement is particularly vital in neurological applications, aiding in the accurate diagnosis, monitoring, and research of brain disorders, thereby significantly contributing to the advancement of medical imaging and patient care. Our proposed method is more generalizable when dealing with multi-acquisition scheme DWIs compared with previous deep learning-based free water elimination methods.

However, the proposed method is also limited to a restricted number of shell configurations, as it depends on the shell configuration of the training data scheme. In our study, we utilize higher b-values of 3000 *s/mm*^2^ as it is used in specific research settings to investigate microscopic features of tissue, such as in studies of white matter microstructure or in more advanced diffusion models. High b-values can be advantageous for capturing complex fiber configurations, like crossing or kissing fibers, and may provide additional information about tissue microstructure. However, these come with challenges like reduced SNR and increased sensitivity to artifacts, necessitating advanced acquisition and postprocessing techniques to mitigate these issues. Typical b-values for clinical applications are usually much lower, ranging from about 500 to 1000 *s/mm*^2^. Besides, not unheard of in specialized research studies aiming to capture finer details of tissue architecture from only 3000 *s/mm*^2^ shells. The high resolution and high SNR properties of the HCP dataset have enabled us to conduct this study. The gap between clinically sufficient and research-oriented datasets can be challenging to the further application of the framework. The introduction of 700 and 1500 *s/mm*^2^ shells will be imperative under the clinical applicable situation.

Additionally, differences in other acquisition parameters, such as spatial resolution, echo time (TE), and signal-to-noise ratio (SNR), can influence the generalizability and performance of the proposed framework. For example, lower resolutions and higher echo times can lead to significant signal attenuation, potentially affecting the free water fraction estimation. Future work should explore approaches to account for these variations systematically, such as through adaptive learning strategies or data harmonization techniques, to extend the applicability of the method across a broader range of clinical and research settings.

The linear decay model employed for b-value mapping offers a simple and computationally efficient solution for harmonizing datasets with different b-value shells. However, this model may not accurately capture the complexity of signal attenuation in certain tissue types, particularly in regions where diffusion is influenced by non-Gaussian behaviors or microstructural heterogeneities. Such simplifications could result in suboptimal calibration, especially when dealing with highly complex tissues. Future investigations could address these issues by employing advanced radial space modeling instead of simple remapping. More potential improvements could include designing an adaptive head instead of the binary coding of shell absence/presence, as introducing more b-values to our proposed method would significantly increase the model parameters.Additionally, incorporating weighted loss functions could improve the model’s ability to handle tissue-specific variations by emphasizing regions with higher free water contamination.

## 7. Conclusions

This study demonstrates that the proposed deep-learning method, trained with heterogeneous multishell data, is capable of estimating tissue volume fractions from measured single-shell diffusion signals. The proposed framework outperforms the conventional method by Pasternak et al., with regularized gradient descent, in correcting single-shell diffusion MRI data. This approach leverages dynamic spherical convolution with data-driven techniques to reliably infer plausible free-water volumes across diverse diffusion MRI acquisition schemes, highlighting its potential for broad applicability in clinical and research settings.

## Figures and Tables

**Table 1:** Comparison of metrics between methods. Both Deep SHORE and our proposed method are trained on a variety of shell configurations to improve the capability of the model. ANN is trained only with single-shell dMRI. The RMSE of *f_fw_* is applied to test the precision of different methods. A Wilcoxon signed-rank test was performed to validate the significance and consistency of model performance, the reference is marked with ★, and all the deep learning method results yielded a *p*-value of less than 0.001.

Category	Method	RMSE of *f_fw_*
Conventional★	Pasternak et al. [6,10,20] w. RGD [28]	3.62 ± 0.44
Deep-learning	ANN [7,37]	3.08 ± 0.31
	Deep SHORE [30]	2.89 ± 0.19
	Proposed	**1.97** ± **0.21**

All metric values are in units of 10^−2^.

**Table 2:** Comparison of RMSE values *f*_*fw*_ for different methods across varying shell configurations. The proposed method demonstrates consistent improvements in free-water fraction estimation over other deep learning frameworks, with tighter variance, indicating higher precision.

Method	Single shell	Two shells	Three shells
ANN [7,37]	2.99 ± 0.35	2.79 ± 0.12	2.41 ± 0.28
Deep SHORE [30]	2.94 ± 0.24	**1.82** ± **0.18**	1.77 ± 0.19
Proposed	**1.89** ± **0.19**	1.85 ± 0.20	**1.63** ± **0.17**

All metric values are in units of 10^−2^

**Table 3:** Comparison of FA (fractional anisotropy) in ROI patches to assess the correction effects of different methods in the CSF-confined ventricles. Values are reported as mean ± standard deviation.

Category	Method	FA (ROI)
Before FWE	–	0.453 ± 0.124
Conventional Method	Pasternak et al. [10] w. RGD [28]	0.498 ± 0.125
Deep-Learning Methods	ANN [7,37] Deep SHORE [30] **Proposed Method**	0.493 ± 0.119 0.501 ± 0.118 **0.508** ± **0.127**
Silver Standard (Upper Bound)	–	0.517 ± 0.124

**Table 4:** Evaluation of model generalizability on the HCP-Aging dataset with direct application to unseen data and fine-tuning on both single-shell and multi-shell configurations. A Wilcoxon signed-rank test validated the significance and consistency of model performance. The reference is marked with ★, and all deep learning method results yielded a *p*-value of less than 0.001.

Method	Training data	Test configuration	*RMSE
Proposed	HCP-YA	1000 (1500), 3000	3.61 ± 0.38
Proposed	HCP-YA	1000 (1500)	3.26 ± 0.36
Proposed (Fine-tuned)	HCP-YA, HCP-A	1000 (1500), 3000	2.22 ± 0.18
Proposed (Fine-tuned)	HCP-YA, HCP-A	1000 (1500)	2.34 ± 0.19
Pasternak et al. w. RGD [28]	N/A	1500	2.89 ± 0.24
Pasternak et al. ★ (Silver Standard)	N/A	1500, 3000	N/A

* All metric values are in units of 10^−2^
